# Global trends in medical adhesive-related skin injury research from 2013 to 2024: A bibliometric analysis via CiteSpace

**DOI:** 10.1097/MD.0000000000046236

**Published:** 2026-04-17

**Authors:** Sha Yan He, Chun Wen Jiang, Hua Lei, Jun Ze Xu, Rong Xiao Mao, Qiong Shi Zhu, Jie Kun Li, Qin Yang

**Affiliations:** a Department of ICU, Chongzhou People’s Hospital, Chengdu, Sichuan, China; b School of Medicine, University of Electronic Science and Technology of China, Chengdu, China; c Department of Nursing, Sichuan Provincial People’s Hospital, University of Electronic Science and Technology of China, Chengdu, China; d Department of Rehabilitation Medicine Center, Sichuan Provincial People’s Hospital, University of Electronic Science and Technology of China, Chengdu, China; e Department of Wound Ostomy Center, Sichuan Provincial People’s Hospital, University of Electronic Science and Technology of China, Chengdu, China; f Department of ICU, Sichuan Provincial People’s Hospital, University of Electronic Science and Technology of China, Chengdu, China.

**Keywords:** bibliometrics, CiteSpace, MARSI, medical adhesive-associated skin injury, visual analysis

## Abstract

**Objectives:**

The study aimed to gain insights into pertinent research and advancements concerning medical adhesive-related skin injuries, thereby enhancing our ability to prevent and manage such injuries effectively.

**Methods:**

Following the technical report ISO/TP21932:2020 on bibliometric visualization specifications issued by the International Organization for Standardization, a comprehensive search was conducted across the database, spanning from 2013 up to July 25, 2024, to investigate the prevalent topics concerning skin injuries associated with medical adhesives. We visualized and analyzed the collaboration network, co-occurrence, co-citation analysis, co-word analysis, bibliographic coupling, burst detection, timeline view, timezone view, overlay maps, and so on using CiteSpace 6.3 tools.

**Results:**

A total of 310 Chinese and 121 English studies were included. The article was distributed across 31 countries, including 31 provinces, autonomous regions, and municipalities, involving 175 domestic research institutions and 164 foreign research institutions. There are 137 Chinese journals publishing articles and 55 English journals publishing articles.

**Conclusions:**

The annual publication volume of medical adhesive-related skin injury-related research, both domestically and internationally, is relatively low. Foreign countries have focused on the research and development of dressings and have refined the research population, such as stoma, tumor, and critically ill patients. After reaching its peak during the 2019 COVID-19 pandemic, the number of publications in China showed a slow downward trend, but those focusing on catheter fixation, pain management, and evidence-based nursing were still slightly higher than those abroad.

## 1. Introduction

Medical adhesive-related skin injury (MARSI) refers to the occurrence of red rashes or abnormal skin symptoms, such as blisters, erosions, and skin avulsion, that persist for more than 30 minutes after the removal of medical adhesives. In July 2013, the World Wound Ostomy and Continence Nurses Association published a statement and consensus on the definition, assessment, prevention, and treatment of MARSI in the Journal of Wound Ostomy and Continence Nurses.^[1]^ MARSI is primarily categorized into mechanical skin injury, tension skin injury, allergic skin injury, and other types (such as maceration and folliculitis).

The incidence of MARSI is approximately 11% to 45%,^[2–5]^ and it varies among different populations. This disease can cause pain, prolong patient hospital stays, increase medical costs, and lead to reversible or irreversible skin damage. With the application of artificial intelligence and virtual reality technology in the medical field, we aim to better understand whether the research hotspots and methods of MARSI differ from previous studies^[6–9]^ in the context of the information age. This study utilizes CiteSpace to explore the current hotspots, status quo, and future research significance of MARSI research, in order to promote clinical understanding of the frontier research on MARSI and better prevent and manage it.

## 2. Methods

### 2.1. Search strategies

Systematic research was conducted across CNKI, WanFang, Chinese Medical Journal Full Text Database, SinoMed, Cochrane, Embase, Wiley, Scopus, Web of Science, and PubMed. The search keywords are as follows: “medical adhesive degloving injur*”/“Medical adhesive skin avulsion*”/“Medical adhesive degloving wound*”/“Medical adhesive related skin injur*”/MARSI/“Medical adhesive-related dermatitis”/adhesive tear injury. AS MARSI is not included in the MeSH terms, the search is conducted using free words and Boolean logic operators.

### 2.2. Inclusion and exclusion criteria

The inclusion criteria are focused on the study of MARSI. The exclusion criteria are as follows: non-Chinese and English literature, animal experiments, and duplicate literature.

### 2.3. Literature screening

**Figure 1.**
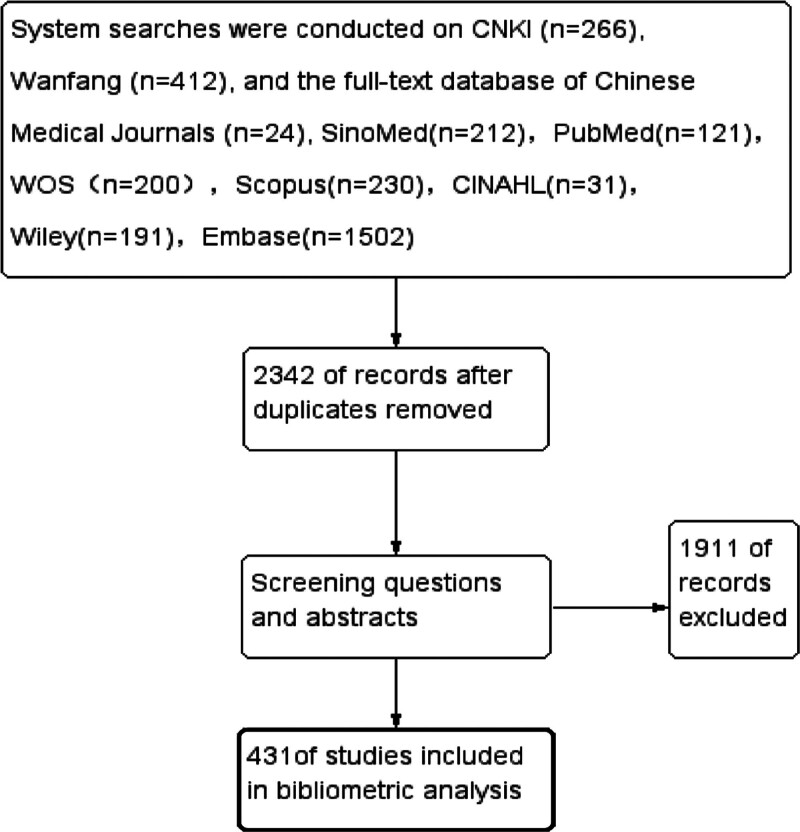
Flowchart of literature screening. WOS = Web of Science.

The preliminary search obtained 3189 pieces of literature, and 431 pieces were finally included in the study after reading the titles and abstracts. Three authors evaluated the paper and applied quality assessments corresponding to different types of literature. The literature screening flowchart is shown in Figure 1.

### 2.4. Research methodology

This study adhered to the technical report ISO/TP21932:2020 on bibliometric visualization specifications issued by the International Organization for Standardization. Utilizing CiteSpace6.3.R1 software (Drexel University, Philadelphia), we conducted a visual analysis of the collected literature on MARSI published between 2013 and 2024, as the definition of MARSI originated in 2013. The literature retrieved from various databases was exported in RefWorks format for data preprocessing. A 1-year time slice was selected, and the node types analyzed included authors, institutions, and keywords. The knowledge network was constructed based on co-occurrence, co-citation, and coupling relationships. Key nodes, such as pivotal literature or high-frequency keywords, were identified through betweenness centrality (BC ≥ 0.1). The quality of network clustering was assessed by combining modularity (modularity *Q* > 0.3) and silhouette values (silhouette *S* > 0.5). Burst detection was employed to reveal the evolution of research frontiers. Network density and clustering coefficients reflected the maturity of the field, while the timezone view demonstrated the migration of research topics.

## 3. Results

### 3.1. Analysis of publication volume

**Figure 2.**
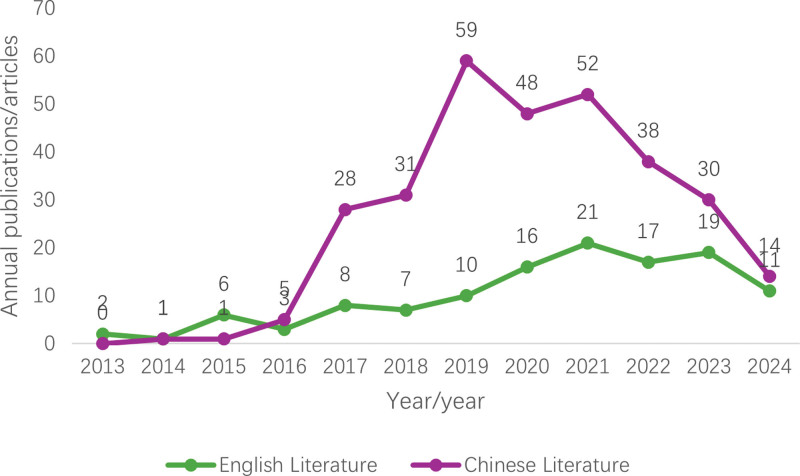
Time distribution line chart of literature (2013–2024).

Of 431 documents, 310 were Chinese documents, and 121 were English documents, of which 16 were Chinese conference documents, 2 books, 15 theses or dissertations, 87 documents had relevant fund project support, 8 foreign review articles, 8 conference abstracts, and 27 articles received relevant funding support. We obtained the number of publications in the past 12 years, imported the data into Excel, and drew a corresponding line graph, as shown in Figure 2. The number of publications in Chinese and English is low. The number of articles in English literature is slowly increasing each year. From 2016 onward, the number of Chinese literature issuances exceeded the number of English literature issuances, and the number of Chinese literature issuances peaked in 2019 and then showed a decreasing trend year by year. However, the overall number of issues was still higher than in foreign countries.

### 3.2. National/regional cooperation network

**Figure 3.**
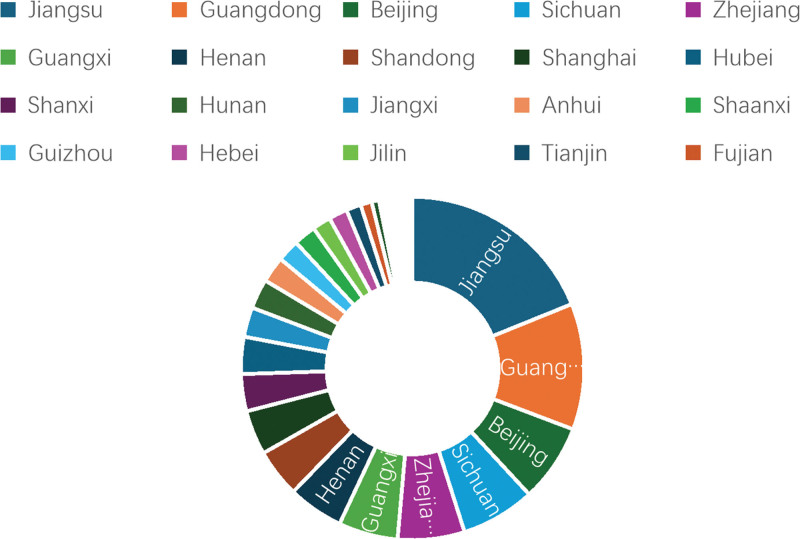
Proportion of Chinese literature published by regions.

**Figure 4.**
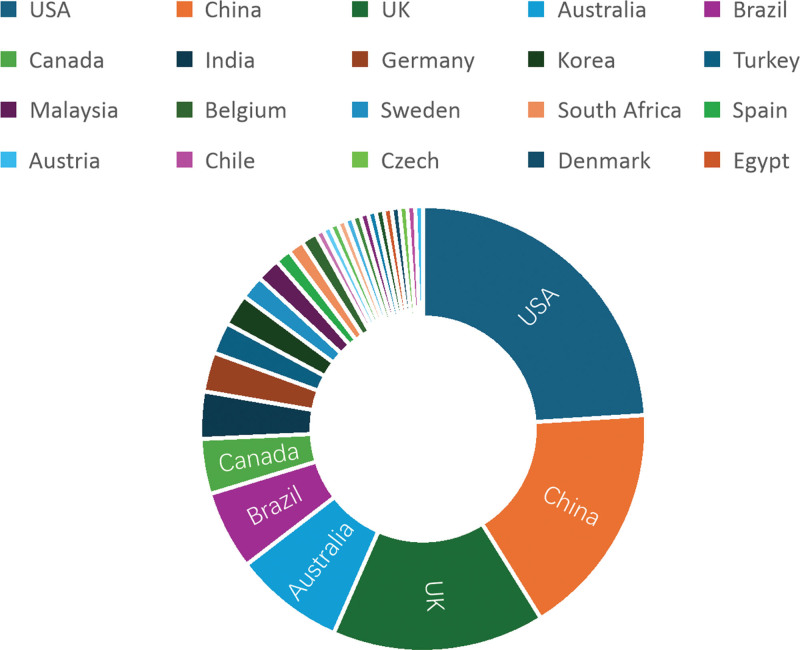
Proportion of foreign literature published by regions.

We counted the regions to which the authors of Chinese literature belong and the countries in which the foreign literature is issued. As shown in Figure 3, the top 5 are Jiangsu (54 articles), Guangdong (34 articles), Beijing (21 articles), Sichuan (20 articles), and Zhejiang (18 articles). As shown in Figure 4, the top 5 are the United States (42 articles), China (30 articles), the United Kingdom (27 articles), Australia (14 articles), and Brazil (11 articles). Among them, the United States accounts for approximately 34.7% of the total number of articles, which is a significant number compared with other countries.

**Figure 5.**
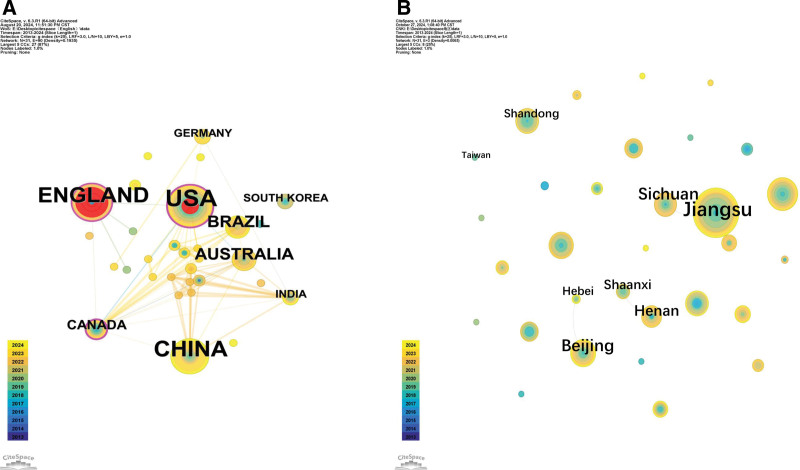
Co-occurrence of countries/origin in English (A) and Chinese (B) literature.

The network node selection Country in CiteSpace yields the cooperation between different countries and regions, as in Figure 5. Node size is positively proportional to the number of literatures, the outer circle represents the betweenness centrality (BD), the thickness is positively correlated with the frequency of literatures, and the connecting selection represents the co-occurrence or co-citation relationship between the 2.^[10]^ Graph A reflects the English literature, with network nodes N = 31 and connecting lines E = 90 (density = 0.1935), indicating a close exchange of collaborations on MARSI research among countries. The top 3 ranked issuances are the United States (BD = 0.34), China (BD = 0.00), and the United Kingdom (BD = 0.19). From the point of view of mediated centrality, the United States is in a higher position in the study of MARSI, with a high number of citations of the literature and high-quality literature, and the issuance of the English-language literature in our country is slightly higher, but the number of citations is not high, and the quality of the literature needs to be further improved; the area highlighted in red is the United States. Quality must be further improved. The red highlighted area represents the highest citation frequency in the UK literature, followed by the United States. Graph B shows the Chinese literature access information, the network node N = 31, and the connecting line E = 3 (density = 0.0065), which indicates that most of the regions in China have researched MARSI, but there is very little cooperation and exchange between the provinces, and the top 5 provinces for the number of studies are in the following order: Jiangsu, Guangdong, Beijing, Zhejiang, and Sichuan, among which the research on MARSI in Jiangsu Province is significantly higher than that in other provinces, and there are cooperative studies with Sichuan Province and Jiangsu Province.

### 3.3. Institutional cooperation network

**Figure 6.**
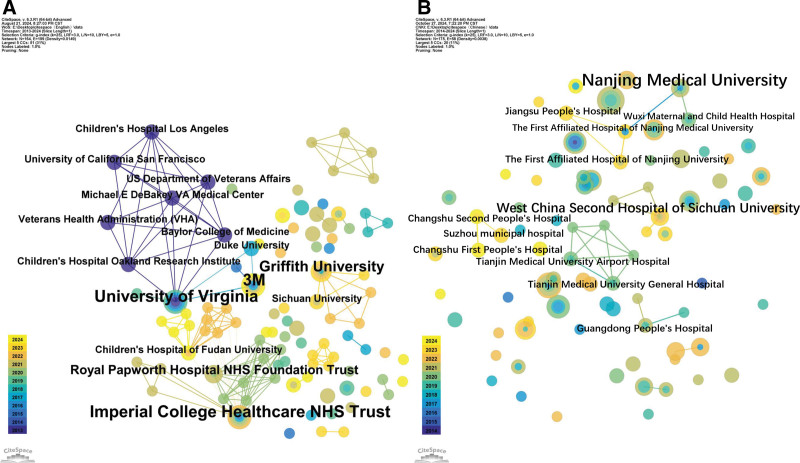
Co-occurrence of English (A) and Chinese (B) issuing organizations.

The cluster analysis of institutions, network segment selection of institutions, and cooperation mapping analysis of sending institutions are shown in Figure 6. The nodes between the lines represent the cooperation between institutions, the line thickness, and the amount of cooperation between the institutions. The number of articles is positively correlated; the thicker the line between the 2 institutions, the greater the cooperation between the number of articles.^[11]^ The thicker the line between the 2 institutions, the more cooperative the publications. The top-ranked major research institutions were the University of Virginia, USA; 3M Company; Imperial College Healthcare NHS Trust, UK; Griffith University and Royal Papworth Hospital NHS Foundation Trust, Australia; Children’s Hospital Research Institute, Oakland, USA; Michael E. DeBakey Medical Center, Virginia; Duke University; Department of Veterans Affairs, USA; Federal University of Minas Gerais, Brazil; and RWTH Aachen University, Aachen, Germany. Figure 6A presents the English-language literature issuing institutions, the co-occurrence map nodes = 164, E = 199, the network structure is dense, there is close cooperation between institutions, and the institutions with strong nodes are the University of Virginia, USA; 3M Company, Imperial College Healthcare NHS Trust, UK; and Griffith University, Australia. University of Virginia, 3M Company, and the Children’s Hospital Research Institute, Auckland, USA, are followed by international collaborations with Imperial College Healthcare NHS Trust, UK; Children’s Hospital of Fudan University, China; Griffith University, Australia; and Sichuan University, China. Figure 6B presents the situation of Chinese literature. The co-occurrence graph of publishing institutions has Nodes (number of nodes) = 175 and E = 58. The total number of Chinese literature publications is larger, but the network structure is relatively loose, and there is a lack of close cooperation among institutions; The leading research institutions were the First People’s Hospital of Changzhou City, Gulou Hospital of Nanjing University School of Medicine, West China Hospital of Sichuan University, and the First Affiliated Hospital of Guangzhou Medical University. The closest collaborators were Nanjing Medical University, the First Affiliated Hospital of Nanjing University School of Medicine, Wuxi Maternal and Child Health Care, affiliated with Nanjing Medical University, and Jiangsu Provincial People’s Hospital.

### 3.4. Author collaboration network

**Figure 7.**
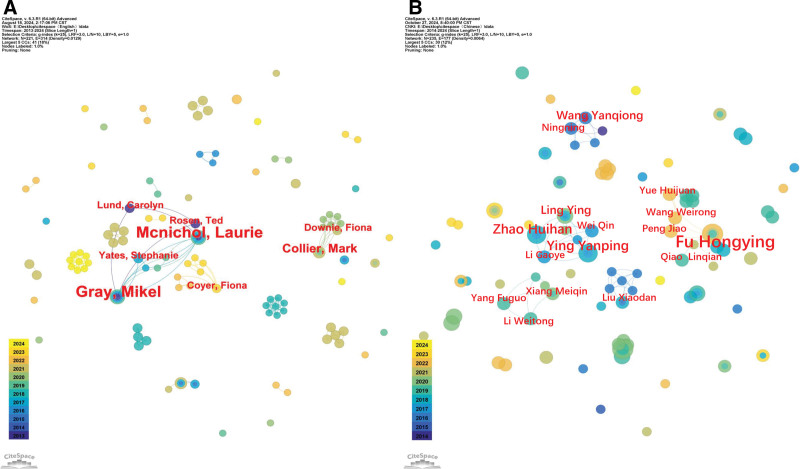
Co-occurrence analysis of English (A) and Chinese (B) authors.

Visual analysis of author collaboration in Chinese and English literature is shown in Figure 7. Figure 7A presents the situation of the English literature, the number of publications tied for first Gray Mikel (n = 5) and McNichol Laurie (n = 5) and tied for second Hitchcock Jan (n = 3) and Collier Mark (n = 3). The authors who had the most collaborations were Gray Mike from the University of Virginia, USA, and McNichol Laurie from 3M Company (USA), and Fu Hongying (n = 5) and Ying Yanping and Zhao Huixin (n = 4) were the second most collaborating authors. There was more collaboration between the authors, such as Fu Hongying from Guizhou University of Traditional Chinese Medicine and Ying Yanping from Guangxi Medical University.

### 3.5. Literature analysis

#### 3.5.1. Co-citation analysis

The statistical ranking of citation counts for English literature shows that the top 10 Chinese articles in terms of citation counts are listed in Table 1. According to Price law, *M* = 0.749 × (Nmax)^(1/2^^)^, to determine the highly cited papers, *M* is the minimum value of the citation frequency of the highly cited papers, and Nmax is the citation frequency of the papers with the highest number of citations. Nmax is the citation frequency of the most cited paper.^[12]^ For English literature, Nmax = 191 and *M* = 71 were calculated. The most cited paper is “Medical Adhesives and Patient Safety: State of the Science Consensus Statements for the Assessment, Prevention, and Treatment of Patient Safety,” published by MCNICHOL L as the first author in 2013.

**Table 1:** Top 10 cited English literatures.

Rate	Title	Year	Country	Lead author	First institution	Citation	JCR	IF
1	Medical adhesives and patient safety: state of the science consensus statements for the assessment, prevention, and treatment of adhesive-related skin injuries	2013	USA	MCNICHOL L	Cone Health System	191	Q2	1.7
2	Overlooked and underestimated: medical adhesive-related skin injuries	2018	UK	FUMAROLA SIAN	North Midlands University	68	Q3	1.5
3	Skin injuries caused by medical adhesive tape in older people and associated factors	2013	Japan	KONYA C	Kanazawa University	61	Q1	3.2
4	Medical adhesive-related skin injury prevalence among adult acute care patients	2015	USA	FARRIS MK	University of Minnesota	54	Q2	1.7
5	Medical adhesive-related skin injury prevalence at the peripherally inserted central catheter insertion site	2018	China	ZHAO HH	Guangxi Medical University	33	Q2	1.7
6	Medical adhesive-related skin injuries and associated risk factors in a pediatric intensive care unit	2019	China	WANG D	Zhejiang University	33	Q2	1.7
7	A randomized and controlled comparison of gentleness of 2 medical adhesive tapes in healthy human subjects	2013	USA	GROVE GL	cyberDERM Institute	31	Q2	1.7
8	Embracing the concept, defining the practice, and changing the outcome: setting the standard for medical adhesive-related skin injury interventions in WOC nursing practice	2017	USA	YATES S	Duke University	30	Q2	1.7
9	Descriptive study of the frequency of medical adhesive-related skin injuries in a vascular clinic	2017	USA	RATLIFF CR	University of Virginia	25	Q3	1.1
10	Medical adhesives-related skin injury in a pediatric intensive care unit: a single-center observational study	2019	Korea	KIM MJ	Pusan University	22	Q2	1.7

IF = impact factor, JCR = journal citation report.

#### 3.5.2. Distribution of journals

The RefWorks format document of the searched Chinese and English literature was used for journal information and sorted using Excel. The journal citation report partition and impact factor values were calculated for the year 2023. The upgraded version of the Chinese Academy of Sciences partition was selected for calculation, and the top 10 journals in terms of the number of articles published are listed in Table 2. In total, 192 journals published MARSI research-related papers, 55 journals published in English, and 137 journals published in Chinese. Among the English journals, J WOUND OSTOMY CONT journal accounted for 18.1% of the articles, Br J Nurs journal for 16.5%, J Wound Care journal for 6.6%, and the number of English-language journals was less and more focused; among the Chinese journals, Electronic Journal of Practical Clinical Nursing accounted for 4.5% of the articles, Journal of General Nursing accounted for 3.9%, and the World’s Newest Medical Information Digest accounted for 3.2%, and the Chinese journals with a large variety of articles were relatively scattered.

**Table 2:** Sorting list of MARSI issuing journals.

Rate	Foreign literature journal	Posting	JCR	CAS	IF	Chinese literature journal	Posting	Type
1	J WOUND OSTOMY CONT	22 (18.1%)	Q2	3区	1.7	Practical Clinical Nursing Electronic Journal	14 (4.5%)	Grade B
2	Br J Nurs	20 (16.5%)	Q1	2区	–	Chinese General Practice Nursing	12 (3.9%)	Grade B
3	J Wound Care	8 (6.6%)	Q3	4区	1.5	Digest World Latest Med Inf	10 (3.2%)	Grade B
4	ADV SKIN WOUND CARE	5 (4.1%)	Q2	4区	1.7	Journal of Nursing	7 (2.3%)	Grade A
5	Nurs Crit Care	4 (3.3%)	Q1	3区	3.0	Chinese Nursing Research	7 (2.3%)	Grade A
6	J Vasc Access	4 (3.3%)	Q3	3区	1.6	Chinese Journal of Modern Nursing	6 (1.9%)	Grade A
7	J Perianesth Nurs	2 (1.7%)	Q2	4区	1.6	Chinese Evidence-based Nursing	6 (1.9%)	Grade B
8	J Tissue Viability	2 (1.7%)	Q1	3区	2.4	Journal of Nurses Training	5 (1.6%)	Grade A
9	Skin Res Technol	2 (1.7%)	Q3	4区	2.0	Contemporary Nurses (First Issue)	5 (1.6%)	Grade B
10	Medicine (Baltimore)	2 (1.7%)	Q2	4区	1.6	Food & Medicine Homology	5 (1.6%)	Grade B

“CAS” stands for the Chinese Academy of Sciences. “Grade A” stands for the highest level in China, with extremely high academic standards and influence. “Grade B” stands for general journals.

IF = impact factor, JCR = journal citation report, MARSI = medical adhesive-related skin injury.

#### 3.5.3. Co-word analysis

**Figure 8.**
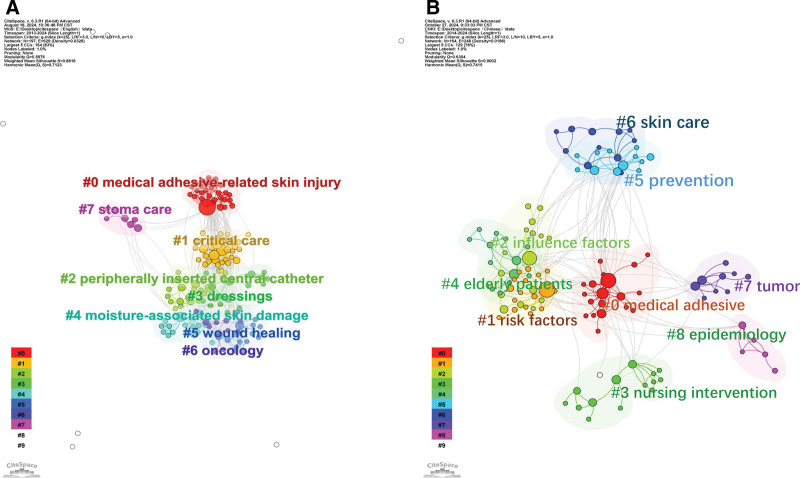
Keyword clustering in English (A) and Chinese (B) literature.

**Figure 9.**
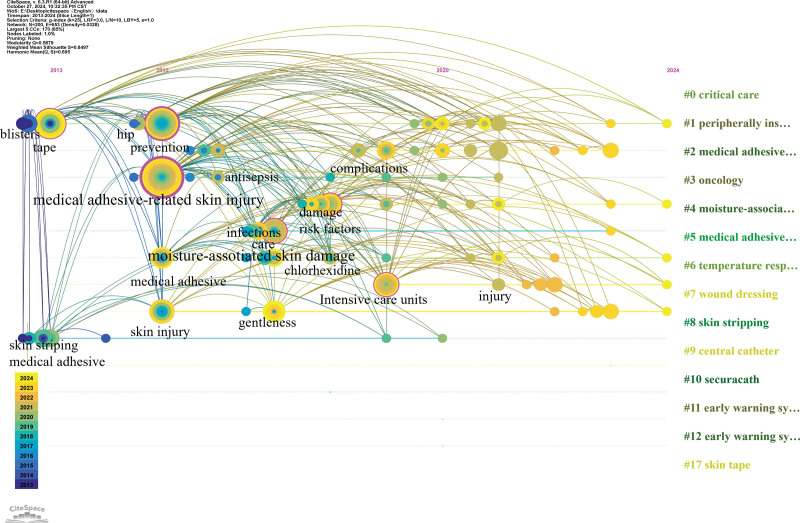
English literature keyword timeline clustering view.

**Figure 10.**
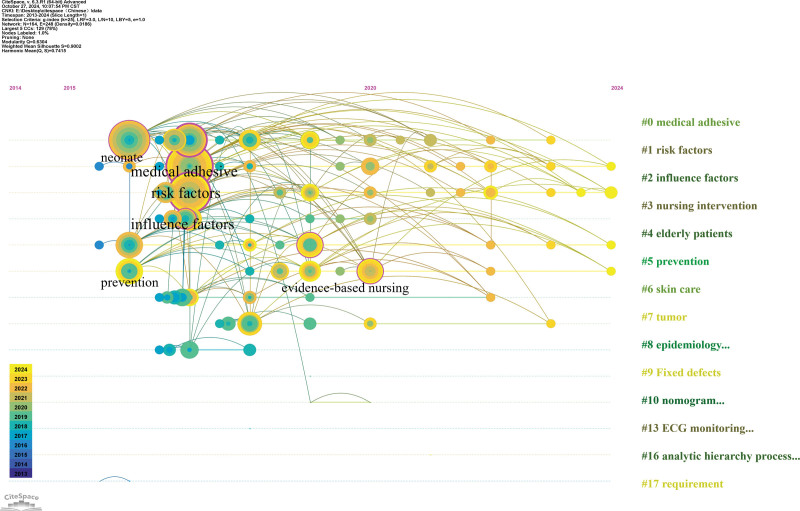
Chinese literature keyword timeline clustering view.

**Figure 11.**
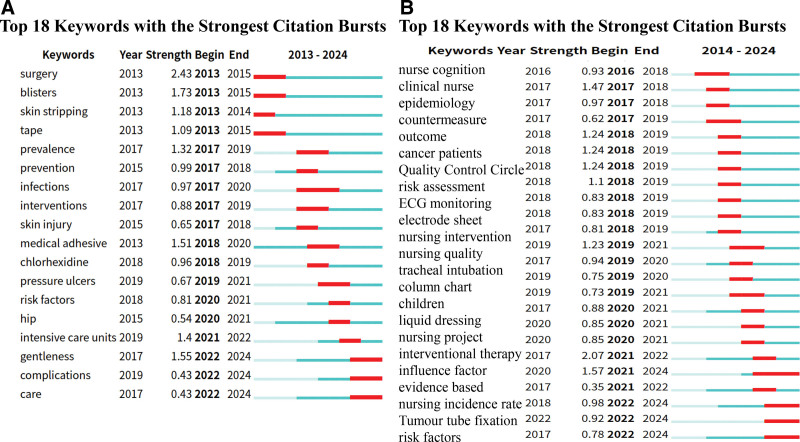
Keyword burst detection analysis of English (A) and Chinese (B) literature.

Co-cited literature was clustered according to similarity, and different clusters were analyzed to determine the core themes of each cluster.^[13]^ We set 1 year as the clustering time to ensure that data integrity did not crop the data. Figure 8 shows the clustering results, modularity *Q* > 0.5 (figure A = 0.5976, figure B = 0.6304), indicating that the literature is divided into reasonable, loosely coupled clusters; the average contour line score is figure A = 0.8816 and figure B = 0.9002, which indicates that the module homogeneity is high. Each cluster label theme was extracted from the keywords. The largest cluster in the foreign literature was #0, with the theme of “medical adhesive-related care”; the themes of the remaining clusters were, in order, “critical care”, “peripheral venous catheter”, “dressings”, “moisture-related skin damage”, “wound healing”, “oncology,” and “stoma care.” The largest clustered themes in the Chinese literature were “risk factors,” “influencing factors,” “nursing interventions,” “elderly patients,” “prevention,” “skin injury,” “tumor,” and “epidemiology.” The above clustering clusters were organized by data provided by keyword mapping through a timeline view (Figs. 9 and 10), reflecting different topics of issuance at different times. As shown in Figure 9, foreign studies have focused on MARSI prevention and risk factors and are now progressively refined to study a specific population, stoma, oncology, catheter placement, and critically ill patients. From Figure 10, it can be concluded that the hotspots of domestic research have changed from risk factors, assessment, prevention, and care in the past to the focus on neonate skin, influencing factors, prevention, and the tendency to use evidence-based, machine-learning prediction, and other methods of research. The keyword emergence analysis is shown in Figure 11, in which graph A is the acquisition of English literature, and graph B is the acquisition of Chinese literature.

#### 3.5.4. Overlay maps

**Figure 12.**
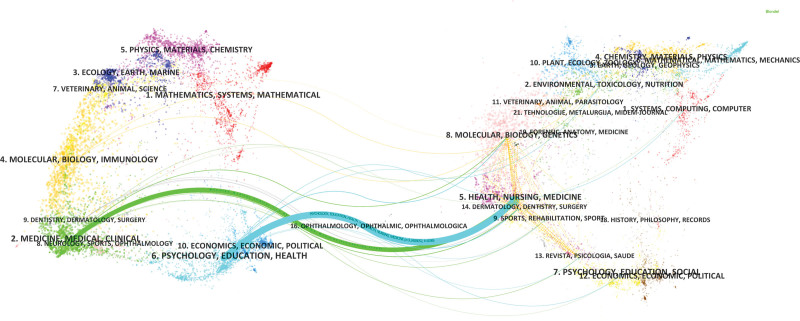
Literature journal stacked map analysis.

Journal overlay maps were analyzed and chosen to conduct a statistical analysis of journals published in English literature. The maps present the main research areas and are divided into 2 parts: the sizing map (left) and the cited map (right). The curves between them are the citation connections; the connected trajectories provide links between the disciplines in the field; the *Z*-score function highlights the stronger, smoother trajectories; and the *f*-value is the citation frequency.^[14]^ Figure 12 suggests that MARSI’s main basic research is in the section on medicine, nursing, and health, and is mainly cited by the sections on medicine and medical diagnostics (*Z* = 2.15, *f* = 477), psychology, education, economics, and policy (*Z* = 3.24, *f* = 666).

## 4. Discussion

### 4.1. Overall situation of domestic and foreign publications

Bibliometric analysis of the retrieved relevant literature on MARSI over the past 12 years revealed that the research attention of foreign scholars on MARSI has been slowly increasing and slightly decreasing domestically, which is still higher than the amount of foreign issuance. In terms of the total amount of literature at home and abroad, the largest number of issuing institutions are in China, the United States, and the United Kingdom. Overseas, the University of Virginia, 3M Company, Imperial College Healthcare NHS Trust, and Griffith University in Australia have conducted extensive research in this area. Domestically, Nanjing University in Jiangsu Province, Fudan University in Shanghai, Guangdong Medical University, Sichuan University, and other medical schools and affiliated hospitals have conducted more research in this field; MARSI has not yet been included in the Mesh Thesaurus, and the total amount of relevant literature published is still relatively small, with the more influential literature mostly coming from research teams in the United States and the United Kingdom.

### 4.2. Latest progress and hot spots at home and abroad

There is a greater emphasis on basic research on developing new types of dressings,^[15–18]^ based on natural biomaterials, silicone, and C14 and C18 copolymers composed of pressure-sensitive adhesive or temperature- and humidity-sensitive new dressings, focusing on different temperatures and humidity skin conditions and different characteristics of the patient population in-depth research, critical patients, stoma patients, and oncology patients.

Domestic research has mainly focused on the causes and management of MARSI, and an expert consensus has been reached. In recent years, the hotspots of domestic research on MARSI have gradually tended to include predictive modeling algorithms, catheter fixation, and pain. Evidence-based methods have been used at home and abroad to explore the relevant research on MARSI, and there have been more domestic than foreign countries, such as meta-analysis^[19–24]^ and evidence summary^[25–29]^ – among them, the use of algorithms^[21,30–35]^ to establish the MARSI prediction model, only applying local area data, the sample size is not large, and can continue to carry out cross-provincial or even cross-country research, to achieve the training model, and at the same time fully validate its external efficacy.

### 4.3. Domestic status and prospects

To date, there are no guidelines for MARSI protection and management in China tailored to the situation of hospitals and the characteristics of the country’s population. However, there is a lack of authoritative and relevant scales for direct assessments. Measurement of MARSI, which leads to a lack of homogeneity of the assessment tools in domestic studies, may ultimately lead to biased results, and existing studies are far from satisfying the requirements for standardized prevention and control of the incidence of MARSI.

A multicenter, multidisciplinary cross-research team has to be established to conduct adequate research on different populations and aspects of related factors and dispositions. Simultaneously, efforts should be made to research and develop new biological dressings that are sensitive to different populations and external environments to facilitate the subsequent development of scientific guidelines and consensus and to establish systematic and comprehensive prevention, control, and management programs. Making full use of the current development of various machine-learning algorithms and information technology, such as artificial intelligence and virtual reality, can improve the management process of MARSI and truly achieve scientific and modern management. Relevant scales, MARSI software, and applications based on various algorithms capable of intelligently identifying staging and recommending treatment methods, effectively reducing the work pressure of nursing staff, scientifically and accurately disposing of the disease, constructing a decision support system, and effectively realizing intelligent and homogeneous prevention, control, and management, to make the study scientific, comparable, and quantifiable, and to further promote scientific control and management, are needed.

## 5. Conclusion

In summary, the current hotspots of MARSI research abroad are mainly for the development of new dressings and the refinement of different characteristics of the population, especially stoma, tumor, and critically ill patients; in China, they are mainly for MARSI pain management, prediction models, catheter fixation, and so on, which lacks homogenized authoritative assessment tools, and systematic and structured quality management processes. We should conduct a classified, phased exploration of the characteristics of different patient populations and seek multicenter and multidisciplinary cross-cooperative research. It is recommended to explore the classification and phasing of different patient populations, seek multicenter and multidisciplinary cross-collaborative research, and strengthen basic research on new biological dressings to ultimately build a comprehensive and systematic assessment, treatment, and evaluation scheme, and issue relevant guidelines suitable for the characteristics of China’s population.

## Acknowledgments

The authors acknowledge all persons involved in the research: Sha Yan He, Chun Wen Jiang, Hua Lei, Jun Ze Xu, Rong Xiao Mao, Qiong Shi Zhu, Jie Kun Li, and Qin Yang.

## Author contributions

**Validation:** Sha Yan He, Qin Yang.

**Visualization:** Sha Yan He.

**Funding acquisition:** Chun Wen Jiang.

**Investigation:** Chun Wen Jiang, Qin Yang.

**Project administration:** Chun Wen Jiang, Hua Lei.

**Conceptualization:** Hua Lei, Jun Ze Xu, Rong Xiao Mao.

**Formal analysis:** Hua Lei, Qiong Shi Zhu, Qin Yang.

**Resources:** Jun Ze Xu, Qin Yang.

**Supervision:** Jun Ze Xu, Rong Xiao Mao, Qiong Shi Zhu, Qin Yang.

**Data curation:** Rong Xiao Mao, Jie Kun Li.

**Methodology:** Jie Kun Li.

**Software:** Jie Kun Li.

**Writing – original draft:** Sha Yan He.

**Writing – review & editing:** Sha Yan He, Qin Yang.
